# Transition of care from hospital to home for older people with chronic diseases: a qualitative study of older patients' and health care providers' perspectives

**DOI:** 10.3389/fpubh.2023.1128885

**Published:** 2023-04-27

**Authors:** Mengjie Sun, Yumeng Qian, Lamei Liu, Jianan Wang, Mengyao Zhuansun, Tongyao Xu, Ronnell Dela Rosa

**Affiliations:** ^1^School of Nursing and Health, Zhengzhou University, Zhengzhou, Henan, China; ^2^School of Nursing, Philippine Women's University, Manila, Philippines

**Keywords:** older patients, chronic diseases, transitional care, health care providers, qualitative research

## Abstract

**Background:**

Transitional care is a critical area of care delivery for older adults with chronic illnesses and complex health conditions. Older adults have high, ongoing care needs during the transition from hospital to home due to certain physical, psychological, social, and caregiving burdens, and in practice, patients' needs are not being met or are receiving transitional care services that are unequal and inconsistent with their actual needs, hindering their safe, healthy transition. The purpose of this study was to explore the perceptions of older adults and health care providers, including older adults, about the transition of care from hospital to home for older patients in one region of China.

**Objective:**

To explore barriers and facilitators in the transition of care from hospital to home for older adults in China from the perspectives of older patients with chronic diseases and healthcare professionals.

**Methods:**

This was a qualitative study based on a semi-structured approach. Participants were recruited from November 2021 to October 2022 from a tertiary and community hospital. Data were analyzed using thematic analysis.

**Results:**

A total of 20 interviews were conducted with 10 patients and 9 medical caregivers, including two interviews with one patient. The older adult/adults patients included 4 men and 6 women with an age range of 63 to 89 years and a mean age of 74.3 ± 10.1 years. The medical caregivers included two general practitioners and seven nurses age range was 26 to 40 years with a mean age of 32.8 ± 4.6 years. Five themes were identified: (1) attitude and attributes; (2) better interpersonal relationships and communication between HCPs and patients; (3) improved Coordination of Healthcare Services Is Needed; (4) availability of resources and accessibility of services; and (5) policy and environment fit. These themes often serve as both barriers and facilitators to older adults' access to transitional care.

**Conclusions:**

Given the fragmentation of the health care system and the complexity of care needs, patient and family-centered care should be implemented. Establish interconnected electronic information support systems; develop navigator roles; and develop competent organizational leaders and appropriate reforms to better support patient transitions.

## Introduction

With the rapid development of population aging, chronic health problems in the older adults are becoming more prominent and the coexistence of multiple diseases is becoming more serious, and the global burden of chronic diseases is increasing dramatically (1, 2). In China, the world's largest developing country, data from the seventh census show that the population aged 60 years and older is about 264 million, accounting for 18.7% of the total population (3), and nearly 3/4 of the older people suffer from one or more chronic diseases (4). Older adults with multiple chronic conditions often have more complex needs, requiring multiple health care providers to provide a wide range of medical and geriatric care facilities in multiple care settings, and often requiring transitions between hospitals and the older adult's own home (5). Several studies have shown that the transition from hospital to home can be a complex, risk-filled process. Approximately one in five patients experience adverse events during this transition, including unplanned readmissions within a month of discharge, medication errors, and even death (6–8). And shorter hospital days may exacerbate this problem and consume even more health care resources, as older adults discharged from the hospital to their homes may have complex health problems, ongoing treatment and care needs (9). Improving the safety and quality of care transitions is an important way to improve patient outcomes and increase the efficiency of health care resource utilization, and has become one of the global concerns (10, 11).

Transitional care is defined as the set of measures taken to ensure that patients receive timely, safe, and continuous health care services, including discharge planning, referrals, follow-up, medication management, and self-education, during the period when patients need to be transferred between sites (hospital-community or hospital-home) or switch between levels of care at the same site (from ICU to general ward) due to changes in their condition (5, 12). *Performance measurement: accelerating improvement:* accelerating improvement identifies patient-centered transition of care from hospital to home as one of the three priority areas for measuring performance measurement (13). Previously Naylor et al. and Coleman et al. reported the significant role of transitional care in reducing readmission rates and improving quality of life in older patients with chronic diseases, among others (14–16), as an important strategy to ensure the safety and meet the ongoing care needs of older patients (17).

However, many problems remain for older adults in the transition from hospital to home, and patient needs are not yet being met (18, 19). With the onslaught of person-centered health care ideology, especially for older patients with higher care needs or complex health problems, health care providers have a responsibility to provide follow-up services and health coaching to bridge system disparities and ensure their continuity of care across levels of care or locations (20, 21). However, the provision of such care depends largely on organizational or system requirements rather than on helping patients or family caregivers achieve individualized goals of care, potentially resulting in patients receiving care that is not aligned with actual needs during the transition or some perceived differences in priorities between health care providers and patients (22, 23). Health transitions in older adults depend on effective self-management, and health care providers need to provide care that promotes sustained and effective self-management in older adults rather than promoting patient dependence. A key challenge in transitional care is the need to provide patients with medical care that is adapted to independent, self-directed needs, i.e., their own perceived, rather than professional-defined needs (24). Although there is a scale developed by Coleman et al. (12) to assess the quality of transitional care services for older patients after discharge, this assessment tool assesses more the overall quality of transitional care and lacks indicators to assess the quality of the structure and process of transitional care services (25). It is also incomplete to understand the patient's perception of transitional care only from his or her perspective. Health care providers (HCP), especially nurses, as the primary implementers and providers of transitional care, interact with other health care professionals to assess patients' biological, psychological, social, and emotional needs, provide community care services including outpatient and home health care, and identify barriers to providing follow-up services and care (26–28). Their perceptions and attitudes about care transitions also influence the safety and quality of transitions (26, 27).

In China, transitional care from hospital to home usually includes verbal health education before discharge and telephone follow-up after discharge, as well as home visits by family physicians and transitional nurses at the request of patients or caregivers. Under a tiered system, family physicians or their teams generally obtain information about and provide follow-up care to older patients during their transition of care from the hospital to their own homes, including medication administration and wound care. However, this model remains challenging due to limitations in primary health care facilities and transitional care hiring staff (29). There is a growing body of qualitative research on the problems and unmet needs of older patients and the ineffectiveness of transitional care (30–32). However, the development of transitional care strategies is influenced by health care resources and cultural differences, and localized policy development needs to be combined with standardization. The perceptions of transition of care among older patients with chronic diseases and healthcare professionals in China have not been adequately described in previous studies. In order for older adults to successfully transition from hospital to home, there is a need to understand the experiences and perceptions of care transitions from both patient and provider perspectives to identify gaps and optimize care transition processes to improve care practices related to this highly relevant area.

The purpose of this study was to explore the perceptions and experiences of transition of care among older patients with chronic diseases and healthcare professionals in a Chinese context to identify potential areas for future interventions to improve the practice of care with this highly relevant transition.

## Methods

### Study design

This qualitative study used semi-structured interviews to explore the perspectives of older patients with chronic conditions and health care providers on transitions of care to understand barriers and facilitators of transitions of care for older patients.

### Ethical approval

The study was approved by the Human Ethics Committee of Zhengzhou University, and verbal consent or written informed consent was obtained from all patients and medical caregivers involved in the study.

### Participants and setting

Participants in this study included older patients and medical caregivers from a tertiary care hospital and a community health facility. A purposive sampling method was used for this study. The inclusion criteria for older adults were: age ≥60 years; coping with at least two chronic diseases (e.g., diabetes, coronary heart disease, chronic obstructive pulmonary disease, stroke); having experienced at least one transfer from hospital to home or undergoing a transition from hospital to home within the past 30 days; and being able to communicate verbally in Chinese (i.e., no language impairment or aphasia). Health care worker inclusion criteria included nurses and general practitioners working in community health care settings involved in the transition of care from hospital to home for older patients with chronic conditions.

### Collection process

Qualitative data collection was conducted between November 2021 and October 2022. Qualitative data collection took place between November 2021 and October 2022. For the recruitment of older adult/adults patients, we explained the purpose of the study and obtained consent from older adult/adults patients 2–3 days before their discharge from the hospital, left their basic information at the time of discharge, and interviewed the patients by phone or at follow-up visits or in the community within 1 month. For health care providers, we contacted the relevant hospital or community agency directors in advance to inform them of the purpose and content of the study, to obtain a list of health care providers willing to participate, and invited health care providers to participate. The interview guide was developed by the researcher team based on the local medical culture and with reference to relevant transitional care researches (33–36). See Table 1 for interview guide. All interviews were recorded and interviewed in Chinese by a team of experienced graduate students, five of whom were Master of Nursing students who had systematically studied and successfully passed the relevant qualitative research program, and two researchers piloted the interview guide to verify its applicability and feasibility. In addition, professionals in the field of qualitative research were invited to revise the interview questions and provide guidance on the interview process. Interviews ranged from 15 to 90 min in length, with notes taken during the interview for initial interpretation. Verbatim notes were taken during the 24 h of data collection. When researchers repeatedly heard similar descriptions in the analysis was no new categories were found that were relevant to the study topic, consider whether the study reached data saturation, and stop collecting data when the research team reached consistency regarding the saturation of the data.

**Table 1:** Interview topic guide of the older patients and HCPs.

**The patient interview guide**	**The HCPs interview guide**
➀ What was your experience with care transitions like?	➀ How did you plan for the older adult's discharge from the hospital?
➁ How do you feel healthcare organizations provide transitional care to older adults?	➁ What were some of the challenges and hardships faced in transition of care?
➂ What difficulties and barriers did you face during your care transition?	➂ How did you cope with them?
➃ How did you cope with the transition of care?	➃ How do you think care could be improved?

### Theoretical framework

To understand the barriers and facilitators that influence older adults during care transitions, this study applied an ecological perspective. This perspective incorporates knowledge from a broader context, including social, economic, political, and cultural factors (37). The theory has been previously applied to studies of transitional care for older adults in the United States (33) and Canada (38). By adopting an ecological or whole-systems perspective to the study of health system issues, interconnectedness across the health care system can be achieved, supporting patients and health care providers to work together to adaptively manage and mitigate challenges in their health care settings (39).

### Data analysis

The data was transcribed and analyzed using Nvivo 12 software and traditional Microsoft Word software. Thematic analysis is used for recording analysis (40). Thematic analysis helps to identify, analyze, and report themes in the data, which is an iterative process where codes and types are compared and analyzed to derive themes (41, 42). Two researchers read the transcripts individually for analysis until a consensus was reached between coding and analysis. The report of this study complies with the Consolidated Reporting Standards for Qualitative Research (COREQ) guidelines (43).

## Results

A total of 20 interviews were conducted between November 2021 and October 2022 with 10 patients and nine medical caregivers, two of which were with the same patient. Ten older patients had a mean age of 74.3 ± 10.1, with an age range of 63–89 years, of whom four were male and six were female. The average age of the medical caregivers was 32.8 ± 4.6, with an age range of 26–40 years, and an average experience of 10.0 ± 6.6, including two general practitioners and seven nurses. Statistical data are detailed in Table 2.

**Table 2:** Demographic characteristics of the older patients and HCPs.

**Demographic information of older patients (*n* = 10)**	***n* (%)**	**Demographic information of HCPs (*n* = 9)**	***n* (%)**
Gender		Gender	
Male	4 (40%)	Male	1 (11.1%)
Female	6 (60%)	Female	8 (88.9%)
Age, mean (SD)	74.3 (10.1)	Age, mean (SD)	32.8 (4.6)
Marital status		Job experience, mean (SD)	10.0 (6.6)
Married	5 (50%)	Profession	
Single/widowed	5 (50%)	General physicians	2 (22.2%)
Living alone		Nurses	7 (78.8%)
Alone	2 (20%)	Place of work	
With spouse	5 (30%)	Tertiary hospitals	4 (44.4%)
With other family	3 (50%)	Community medical institutions	5 (55.6%)

### Attitude and attributes

Attitude and attributes are both important facilitators and barriers to transition of care for older adults. The active participation of some older adults in care transitions and their confidence that they can have a bright future at home is a sign of their positive attitudes.

“I came home without any problems, and if it wasn't for the coronavirus, I would have been out traveling again, haha.” (P8)

“The community institution is very close to my house, a few minutes walk away … The doctors and nurses also call me regularly, the community often conducts medical consultations, there are doctors' phone numbers saved, and we have a family bed …Home will make I feel safer.” (P3)

Despite the fact that the conditions at home are not as good as those in a skilled nursing facility, older people still choose to return to their families. This has to do with the influence of traditional Chinese culture, where the idea of filial piety and raising children for old age is deeply rooted.

“I suggested a referral to a professional nursing home, and he refused.” (HCP4)

“I have children, I am not going into a nursing home. To let the older people live in a nursing home is to have children who are not filial and will be spit upon by others.” (P9)

When certain symptoms appear, some older people participants will first manage the symptoms based on their past experiences, and then seek medical help as a last resort when their self-tries fail. This is related to the financial income of the der persons, the taboo to seek medical help, and the fear of “covering their ears.”

“Nowadays, older people are bound to live and die with chronic diseases, and it makes sense that the old saying ‘a long illness becomes a good doctor.' Like I've been diabetic for so many years, I know what to do when my blood sugar is high … I think I'm capable of dealing with some unexpected situations and don't have to come to the hospital for everything.” (P7)

“Wheezing when you hurry to sit down and rest … breathing poorly the first reaction should be to find medicine and oxygen equipment … when taking medicine also does not work, I can only go back to the old place again, although I reluctantly hospitalized, but no way I do not want to die yet.” (P4)

“They always endured until they could not endure before they came to the hospital, I asked why they did not come earlier, some of them would justifiably reply: I used to come over like this, it is not a very serious matter, it became complicated to come to the hospital, I do not want to come to the hospital … seems very open-minded, but in fact the fear of the hospital, which has to do with their stubborn perceptions, which is hard to change.” (HCP1)

To further achieve independence at home after discharge from the hospital, some participants learned new skills, including blood glucose monitoring, injections, and oxygen, complex medication management, and rehabilitation-related care, in addition to having positive thoughts, in an effort to improve self-management, learn self-care skills, and take personal responsibility for self-care.

“There are many people who have had their hands and feet cut off because of diabetes. Our neighbor, who had a bad heart and diabetes, cut off a toe, and the palm of her foot (wound) did not grow easily, it festered, and (the surface of the foot) ran through. It will bring trouble to my son, I usually pay more attention now, I don't eat anything sweet!” (P3)

“You are the first person in charge of your health, don't think about other people reminding you, relying on others won't work.” (P9)

Yet transition is a process of transformation across organizational settings, and as older adults faced a transition from a medical facility to an unstructured home environment, some participants expressed a sense of uncertainty and concern about in-home care. They exhibited fear, depression, loneliness, and even despair, all of which are barriers to transitional care. This negative perception of transition was associated with symptoms that had not yet disappeared, negative experiences of past transitions, and knowledge skill gaps in dealing with physical changes and managing adverse reactions.

“I don't want to be discharged home, I don't feel cured, I can't talk or walk easily now, what if I fall, what if a blood vessel ruptures again? Will I still be able to run away from Hades.” (P9)

### Better interpersonal relationships and communication between HCPs and patients

The conventional wisdom is that discharge from the hospital represents the end of the relationship with the hospital. During the care transition journey, patients fear abandonment by their medical caregivers due to unresolved illness, desire lasting interpersonal relationships and connections with them for follow-up appointments and timely follow-up care, and affirm the role family caregivers play in facilitating and coordinating communication between each other.

Older adults require frequent travel between the hospital and home due to recurring, migratory non-healing chronic conditions. Some participants emphasized the desire to continue contact with medical caregivers after discharge from the hospital so that problems can be responded to quickly.

“It would be nice to have a contact number for the doctor or nurse so that I can call if there is a problem.” (P1)

“Provide the phone number of the department so that when I have a problem it can be dealt with promptly, preferably the supervising doctor.” (P6)

Several participants reported the importance of maintaining long-term, trusting relationships with medical caregivers. There is no doubt that this provides a better opportunity for medical staff to help patients transition as well.

“I come to Dr. Jia every time I have a problem, I trust her, she understands my situation, I don't reject a new doctor to reach me, but the new doctor prescribes medication that is not consistent with what I had before and I can't maintain trust in it ….” (P3)

“I can't know every patient like the back of my hand, it takes time and frequent contact, and when the conversation is accurate and I tell her [the patient] background, her condition, I think she will open up.” (HCP3)

Communication between the HCP and the patient was seen as a barrier when medical diagnoses or discussions of conditions were not delivered in a way that the patient could understand or when too much medical jargon was used, and limited the trust and relationship that some older participants had with the medical staff.

“… Not everyone is a medical student, when they say medical terminology words, I know they are talking about my condition, but it sounds as if it is a pipe dream, they tell me that a certain indicator on my labs is high and I might have a sudden brain attack, but what do I know? ….” (P6)

In addition, the tone and attitude of care providers' speech and workload were daunting to patients.

“The doctors and nurses are very busy, taking time out for meals, in and out of … I know they are here, I made several trips to the doctor's office and couldn't find them ….” (P4)

“I went for a follow-up appointment and he looked at my report card and simply said two words and called the next person without even looking up at me … I understand that they are busy, but I felt lost inside, like an assembly line job with no emotion.” (P10)

It is clear that older adults and HCPs recognize the important supportive role that family caregivers play in building, maintaining, and communicating with each other and even in the transition of care for older adults. Older adults who are confused about discharge instructions and follow-up plans due to memory, comprehension, or mobility limitations require family caregivers to be involved in discharge education or counseling about medical decisions and matters. Whereas communication and contact among healthcare professionals is not real-time and fluid, family caregivers facilitate the transfer and sharing of patient care information among different healthcare providers.

“It's better to have a family member there who will provide additional information, such as previous visits, medication information and test reports, etc. The more detailed I know, the better I can treat the patient.” (HCP4)

An interesting phenomenon is the transformation of family caregivers from supportive supporters to surrogates and advocates for care decisions, and the closeness of the relationship between health care workers and family caregivers even more than patients, which are mutually understood and commonplace.

“When I had mobility problems, I would ask my children to contact Dr. Jada by phone, and they had WeChat between them for easy contact.” (P3).

“I would explain some precautions, let's say you have diabetes and you need to control … on your diet, and the patient would have family members come along to participate….” (HCP6)

“It's important to educate the caregiver about health and the family needs to understand which symptoms are present and they need to come to the hospital quickly and not delay at home.” (HCP3)

In contrast, for older adults who live alone or not with a younger caregiver, health care providers are selective in their coverage and patient education, which is related to their level of health literacy and acceptance of health education.

“Explaining so much they (he and his older partner) can't understand. I would prefer to have the younger caregiver around, and they are sorry it's not mandatory. A lot of times I will say something in layman's terms and in layman's terms depending on his education level, so that you don't intermittently ask you questions when you tell him something to do.” (HCP2)

### Improved coordination of healthcare services is needed

Care coordination refers to the coordination of care providers between different health care facilities or within a health care facility. A coordinated health care delivery system is an important facilitator of care transitions. Poor coordination among health care providers leads to difficulties in effective communication and information transfer, and thus inconsistent and underserved care delivery.

Older adults have detailed records of their visits during hospitalization, but after discharge patients have to repeat their health information and medication details to other health care providers, and even repeat some medical tests. This caused dissatisfaction and frustration among patients and health care providers, and expressed the need for a well-developed electronic system to avoid duplication or inaccurate transmission of verbal information to patients as well as to facilitate advance knowledge of patients, facilitate knowledge transfer of diseases, and what steps to take to complete care planning.

One participant indicated,

“When you leave the hospital, the hospital should pass on the information to community agencies. This is correct. But in reality, the hospital doesn't do that, and the community agency doesn't know that you've been discharged; they don't know anything. You had to repeat the process… They didn't do a good job of bridging the gap.” (P2)

“My CT report should be shared between hospitals instead of wasting money on re-testing.” (P5)

“We belong to different systems and we can only see information about the patient's treatment within the same tissue, and they (patients) also often complain that we have taken imaging tests at other hospitals and suspect that we are getting money from them …, when in fact we don't get their details.” (HCP3)

Increased collaboration among care providers can be beneficial to patient transition. Older adults and HCPs expressed the need for a role model to navigate the health care system during care transitions. Patients don't understand how the health care system works, and they want a dedicated person to interface with community agencies or family physicians for information prior to discharge and to be able to contact for help if they are overwhelmed after discharge.

“I hope the hospital and community [agencies] complete the docking … for good symptomatic management,” adding further, “There is no point of contact between the clinical staff and the community health care providers, and our community is not affiliated with this hospital. The hospital should have a dedicated person to help us find the docking community organization.” (P6)

Although medical caregivers also agreed with having coordinators to navigate care transitions, there was disagreement about who should serve. Most patients felt that there should be their own supervising physician or nurse who was familiar with them. Nursing staff, on the other hand, felt that a dedicated person should be set up to coordinate and that it should not be the busy front-line clinical staff doing this job.

“… In the hospital it would be just treating acute symptoms and more importantly home regimen … In this gap, despite the follow up, it does not seem to meet the patient's needs….... We should create a professional team with dedicated staff to do this and create a docking organization between the department and community organizations or home care. Let's say a patient is discharged from our ward and we notify him a day in advance and say come on, here's a patient who needs to be discharged ….” (HCP8)

In reality middle-aged and chronically ill patients often have more complex care needs, fragmented health systems and limited availability of resources, and health care workers have to make more efforts to coordinate care transitions and rely more on positive personal traits. Yet some participants indicated that imperfect financial incentives and regulatory measures may further increase the care coordination gap.

“… on care transition coordination is more on the individual, there is no mandatory system that says you have to do it, I do the work and I don't have any performance income, and I would struggle with guilt and busy work if I didn't make the effort for it.” (HCP9)

### Availability of resources and accessibility of services

Older patients are discharged from the hospital with unresolved illnesses and high levels of need for follow-up care information and resources during the transition of care, and then there are systemic barriers to care transitions due to the lack of a holistic transition of care plan in the health care system, resulting in a lack of available medical resources and expression of service accessibility after discharge.

Returning home represents the end of the medical journey, and participants report a lack of follow-up ongoing follow-up and a sense of isolation and abandonment in the transition of care.

“No one cares about you after you leave the hospital, the doctors don't call, the nurses don't,” the participant further explained, “When you go home you are on your own, you don't know who to call if something goes wrong.” (P2)

For participants in remote or rural areas, they reported that there were no available medical teams and resources, especially when mobility was limited and transportation was difficult, and more effort had to be made to ensure the transition.

One older participant said

“It was a particularly small clinic, with only one staff member doing both doctor and nurse work. And there were no rehabilitation facilities. I had no choice but to municipal hospital, the one side of my body had paralyzed, which was troublesome for me and required a lot of stuff to bring, just like moving.” (P9)

Although providers could identify patients' needs, staff shortages prevented them from providing appropriate care.

“Caregivers ask us for home infusions, and I know that's their expectation, but it's unrealistic.” (HCP3)

In addition, there is limited training on mental health issues and needs of older adults, and health care providers are more focused on addressing somatic symptoms.

“… he appears mildly depressed, we'll just have to comfort and reassure, I can't inject drugs into his psyche.” (HCP7)

And with the pandemic outbreak of the coronavirus epidemic, the implementation of some prevention and control measures has made follow-up appointments and home visits challenging.

“My neighbor he got infected and the community told me I needed to stay out for 7 days, I was supposed to go to the hospital to review my heart, but there was no way I could do that, I had to wait until the unblock.” (P5)

Healthcare organizations have introduced Internet-based digital communication technologies to protect staff safety and reduce face-to-face care services, but the availability of supportive resources and the accessibility of services remain limited, which may be related to the e-health literacy of older adults and the infrastructure of community agencies.

### Policy and environment fit

China's 13th Five-Year Plan emphasizes continuity of care, although the state has introduced a series of incentives to facilitate transitions of care for older adults. However, current policies still lack specificity and certainty, making transitional care for older adults fraught with opacity and immaturity.

“I know the Health Care Commission has a policy, but that's too big and I need more detailed rules to implement ….” (HCP5)

Stable, competent leaders play an important role in policy planning and guidance for care transitions for older adults.

“The central government sets the documented policy and the hospital has to implement it, but you can't implement it blindly, you have to understand the plight of older adults, and the key is to rely on a handful of people, depending on the ability and also the level of dedication.” (P6)

In addition, one nurse expressed the need to consider the medical cultural environment when implementing transitional care.

“Transitional care is in a limbo, many aspects are not perfect, our health care system is different, we can't simply copy foreign countries, the care environment we are in is very different ….” (HCP8)

## Discussions

Progress in transitional care has been made in China since 2011.But the continuum of management of older patients with chronic conditions after discharge from hospital remains fragmented (44). To improve continuity of care for older adults with chronic conditions, we explored older adults' and health care providers' perceptions and experiences of transition care and identified five themes: attitudes and posture, better interpersonal relationships and communication between health care providers and patients, need for improved care coordination, availability of resources and accessibility of services, and policy and environmental adaptations. These themes are often both barriers and facilitators that affect the care of older adults in transition. For example, at the individual level, positive beliefs of the older person and coping strategies to address stressors between the individual and the environment are considered valuable; at the interpersonal level, relationship continuity based on trust enhances patient confidence in transition; and at the policy level, competent organizational leaders are important enablers of transition care development. However, there appears to be a disconnect in post-hospital follow-up care support. For example, there is a lack of available medical resources, a lack of clear accountability and incentive systems, and neglected cultural attributes. Among these factors, consistent with the broader international literature, are family caregiver advocacy, care coordination, and relationships between patients and providers (33, 34, 45).

Our study found that patients' personal attributes and posture play an important role in care transitions. Complex emotional responses in older adults are associated with confidence in home care, coping with outcomes, and ongoing health status. This is consistent with studies Hestevik et al. (24) and by Dolu et al. (34), in which older patients are experiencing insecure transitions due to symptoms that have not disappeared after discharge, personal loss of control over their lives, and self-management difficulties, where safety and stress are intertwined in care transitions. Despite the unstructured nature of the home environment, influenced by filial ideology, older chronically ill patients choose to transition at home and struggle to find ways to take control of their lives and coping strategies to stay at home for as long as possible, which is a sign of both positive attitudes and barriers to the use of formal care for older adults. Coping strategies emerge under the influence of personal beliefs, and coping outcomes in turn influence patients' experiences and confidence in care transitions. According to Lazarus, the primary function of coping is to address interpersonal stress and emotional regulation by doing something to address the person's environment, and people who master a sense of control may be better able to cope with their environment and stress. In our study, the coping styles of older patients with chronic illnesses included symptom management according to pre-existing experiences and learning self-care skills, which are positive, problem-oriented coping strategies. This is consistent with Backman et al. (33) and Allen et al. (35) studies, where older adults developed self-management skills by actively adapting health behaviors and skills to better manage their illness. This is a result of the free choice of people with chronic conditions to decide how to live with dignity and cope with their current situation. This has clinical implications in a system that promotes the acquisition of feelings of helplessness and dependency in older adults who use health care services. For example, health care professionals may listen to patients' stories to better understand the personal meaning and different ways that people with chronic illnesses cope with daily life, helping them to identify when their coping style may not be in their best interest or the transitional impairment it causes. Thus, there is a need to see others as “people” rather than as groups and “objective” data, and to be sensitive and responsive and adaptable to their specific personal characteristics in transitional situations.

Patient and family involvement in care is central to global health care. We found that both patients and healthcare providers recognize the value of including family caregivers in the care transition circle, which is consistent with previous studies (46, 47). Prior literature has focused on the need for patients and families to receive information (48), and we also highlight the important role family caregivers play in providing information to healthcare providers, which supports the study by Baxter et al. (21). Family caregivers providing information to medical staff to proactively bridge system gaps is an effective way to support safety and quality of care, especially in a context of dysfunctional communication systems and changing organizational structures (49). Furthermore, in addition to patient and family caregiver efforts in care transitions, health care providers should abandon their paternalistic medical authority style (24), such as the use of professional jargon and distant attitudes when talking (50). Thus, for health care providers, care decisions need to be made based on the background, preferences, and values of older adults (51), which is consistent with the basic principles of patient-centered care (32). Otherwise, care can become authoritarian, invasive and oppressive to the patient, and detrimental to the establishment of a trusting relationship.

Care coordination is an area of current focus for improvement to achieve cross-organizational and cross-professional integration. In our survey, older adults described disruptions in care delivery when transitioning from hospital to home, and disconnects between tertiary hospitals, community-based organizations, and home were associated with the occurrence of adverse events, and medication errors (52, 53). In line with Baxter et al. (21), technology can be used as a way to improve safety during the transition. Therefore the use of relatable information support systems in holistic care is essential to support the seamless transfer of electronic medical records and prior visit records between hospital, community, and home care for care coordination (54). In addition, consistent with previous research, the navigator role needs to be introduced in “siloed” systems as a link between patients, informal caregivers, and health care providers during transitions in the health care system (55). The navigator typically advocates, empowers, motivates, and helps to identify, anticipate, and mitigate barriers that patients encounter during transitions of care, reducing readmission and emergency department return rates (56, 57). However, it is worth noting that whether clinical frontline staff or specialized agency staff navigate care transitions more cost-effectively and economically needs to be further explored.

The imbalance in care resources during the transition from the hospital to the unstructured home and the poor availability of services do not meet the needs of older patients with chronic diseases for home rehabilitation. Previous studies have similarly shown that this unbalanced relationship may affect the successful achievement of established care goals. In China, universal health coverage has been actively pursued after the reform of the healthcare system, but due to the size of the population and geographic distribution, significant differences in healthcare resource availability, staffing, and accessibility of services between urban and rural areas have been identified as barrier factors to transitional care (36). It can be a challenging task for care providers to prioritize their needs and make decisions. Some health care services and medical teams, require patients to travel to the inner city to receive care. And financial subsidies are not available to cover the various non-direct care-related costs that older adults incur to access health care, so there may be additional structural financial barriers to accessing care for older adults living in regional and rural areas. Health systems are the ultimate drivers of care practices for patient populations. In the West, the practice of transitional care is dependent on the appropriate medical resources and health care systems. The Affordable Care Act in the United States (58), the evolution of government-funded outpatient and subacute care models in Australia (59), and the development of the Medicare Trust model in the United Kingdom (60) have made transitional care for older adults cost-effective and cost-efficient. The future Chinese healthcare system may need to consider transitional care for older adults and make appropriate adjustments.

With the COVID-19 pandemic, limited contact between older adults and their families and healthcare providers has facilitated the widespread use of telemedicine (61, 62). Despite the fact that older adults are largely digitally illiterate, studies have found that older adults find tele-rehabilitation acceptable, improving access to health care (63). This is especially true when telemedicine offers the possibility of medication reconciliation and regular visits for patients who would not otherwise receive post-discharge follow-up or who have mobility and transportation limitations, as well as frailty. However, the first thing that needs to be addressed are the barriers to acceptability and implementation of telemedicine for older adults in order to improve the achievement of the target system. Otherwise, these digital interventions may not only exacerbate any problems that older people already face when trying to access health and social care services. It could also, in turn, affect the workload of primary care, and health care providers must take greater responsibility for ensuring that this important population receives the care they need (64).

In addition, our findings suggest that effective care transitions require strong leaders in addition to organizational and system changes. This is consistent with previous research that having a person or team within an organization that supports transitional care delivery can be a powerful catalyst for change (65).

## Limitation

The study conducted interviews with older patients with chronic conditions and frontline health care providers, and future interviews could be conducted with health system leaders and older family members with chronic conditions about their experiences during the transition from hospital to home care and their perceptions of improving the transition of care process. The final study was limited to one city in central China and cannot be fully transferred to other urban settings; more research is needed to determine the generalizability of the results to develop and formulate patient-centered transitional care interventions.

## Conclusion

We explored older adults' and health care providers' perceptions of care transitions with enabling factors including positive personal attributes, coordination of services across organizational settings, effective communication among health care providers, provision of resources, and policies that are compatible with cultural environments. Identifying these barriers and enablers provides an opportunity to improve the complex needs of patient care in a fragmented health care system while maintaining a patient and family focus. This provides strategic options for improving access to care in complex systems.

## Data availability statement

The original contributions presented in the study are included in the article/supplementary material, further inquiries can be directed to the corresponding author.

## Ethics statement

This study was approved by the Ethics Committee of Zhengzhou University in China (approval number: ZZUIRB2021-78). The patients/participants provided their written informed consent to participate in this study. Written informed consent was obtained from the individual(s) for the publication of any potentially identifiable images or data included in this article.

## Author contributions

MS conceived the study, collected the data, and conceived the first draft. MS and JW developed the interview guide and performed the transcription and data analysis. YQ refined the theme, participated in the writing of some of the first drafts, and was responsible for the revision and finalization of subsequent manuscripts. LL approved the guide. MZ and TX provided support. LL and RR performed quality control of this study and critically revised the manuscript for important intellectual content. All authors have read and approved the final manuscript.
